# Mammalian Sirtuins and Their Relevance in Vascular Calcification

**DOI:** 10.3389/fphar.2022.907835

**Published:** 2022-05-23

**Authors:** Xinyue Pan, Caixia Pi, Xianchun Ruan, Hanhua Zheng, Demao Zhang, Xiaoheng Liu

**Affiliations:** ^1^ State Key Laboratory of Oral Diseases, West China Hospital of Stomatology, Sichuan University, Chengdu, China; ^2^ West China School of Basic Medical Sciences and Forensic Medicine, Sichuan University, Chengdu, China

**Keywords:** cardiovascular disease, vascular calcification, sirtuins, calcium phosphate, histone deacetylase

## Abstract

Cardiovascular diseases are a group of diseases with high morbidity and mortality that affect millions of people each year. Vascular calcification (VC) is an active process that involves the mineral deposition of calcium-phosphate complexes. VC is closely related to cardiovascular diseases, such as hypertension, heart failure, and calcific aortic stenosis, and is a type of ectopic calcification that occurs in the vessel walls. The sirtuins (silent mating-type information regulation 2; SIRTs), are a family of histone deacetylases whose function relies on nicotinamide adenine dinucleotide (NAD+). They have non-negligible functions in the regulation of energy metabolism, senescence, apoptosis, and other biological processes. Sirtuins have important effects on bone homeostasis and VC processes that share many similarities with bone formation. Sirtuins have been confirmed to deacetylate a variety of target proteins related to the occurrence and development of VC, thereby affecting the process of VC and providing new possibilities for the prevention and treatment of cardiovascular diseases. To facilitate the understanding of vascular calcification and accelerate the development of cardiovascular drugs, we reviewed and summarized recent research progress on the relationship between different types of sirtuins and VC.

## Introduction

As a type of ectopic calcification, vascular calcification (VC) is the process of mineral deposition, in the form of a calcium-phosphate complexes, in vessel walls. VC is closely related to the occurrence of cardiovascular diseases, such as hypertension, heart failure, and calcific aortic stenosis, which is one of the major causes of human death (Johnson et al., 2006; Cano-Megías et al., 2019). VC is a complex event that is mediated by different types of cells and active processes that are similar to those involves in ossification and bone formation. It is usually not determined by a single factor, but rather, is influenced by multiple factors, including genes, the environment, and blood vessels (Villa-Bellosta et al., 2011). According to the location of mineral deposition, VC can be divided into intimal and medial calcifications. Intimal VC is associated with lipid deposition, inflammation, and necrosis. It often occurs in large arteries, is linked to obstructive arterial disease, and is usually associated with atherosclerosis. The most characteristic feature of medial calcification is the transdifferentiation of vascular smooth muscle cells (VSMCs) into osteoblasts, from a synthetic phenotype to a contractile phenotype. This usually results in reduced blood flow and often occurs in patients with chronic kidney disease (CKD), diabetes, osteoporosis, and hypertension (Wu et al., 2013; Lee et al., 2020a; Singh et al., 2021). VC is a threat to human health, but effective drugs that inhibit or reverse the processes of VC are currently lacking.

Mammalian sirtuins contain seven members: SIRT1-7. They are evolutionarily conserved from bacteria to eukaryotes, with catalytic sites formed by the hydrophobic channel between NAD + binding Rossmann folding domain and Zn2 + binding domain, and most have been confirmed to be protein deacetylases whose function relies on nicotinamide adenine dinucleotide (NAD) (Michan and Sinclair, 2007). According to sequence similarity, mammalian sirtuins are divided into at least four classes: classes I–IV. Class I contains SIRT1-3, SIRT4 belongs to class II, SIRT5 is in class III, and SIRT6 and SIRT7 belong to class IV (Frye, 2000). The sirtuin distribution within cells can differ depending on the protein. The three nuclear proteins are SIRT1, SIRT6, and SIRT7. SIRT3, SIRT4, and SIRT5 are localized in the mitochondria, an organelle in which many important metabolic processes occur. SIRT2 is the only sirtuin that is mainly distributed in the cytoplasm (Michishita et al., 2005). The most studied enzymatic activity of the sirtuin family is deacetylation, represented by SIRT1. However, some of the sirtuin family members have weak deacetylase activity and other enzymatic activities (Michan and Sinclair, 2007). For example, SIRT5 was found to remove succinyl and malonyl from protein lysine residues (Du et al., 2011). Sirtuins have important biological functions and participate in many biological processes, including cell proliferation, apoptosis, DNA repair, and cell metabolism. They are also highly associated with many pathologies, such as neurodegenerative diseases, cardiovascular diseases, and cancer (Carafa et al., 2016).

Among the sirtuin family members, the most abundant one, SIRT1, is widely believed to be closely related to and play a significant protective role in VC (Lu et al., 2020). Moreover, SIRT3 and SIRT6 have been shown to play significant roles in protecting the vasculature against atherosclerosis (Sosnowska et al., 2017). Several signaling pathways connecting sirtuins and VC have been identified (D'Onofrio et al., 2018). However, research on other sirtuin members, especially SIRT5, is lacking. In recent years, the link between sirtuins and VC has been a research focus, and new discoveries have been reported. In this review, we summarize the new research results of SIRT1-7 connected with VC in the past 5 years, with the aim of providing researchers help and inspiration to find effective VC drugs.

## Vascular Calcification

### Classification of Vascular Calcification

According to the location of mineral deposition, VC can be mainly divided into intimal and medial calcifications. Different types of VC have different characteristics.

**FIGURE 1:**
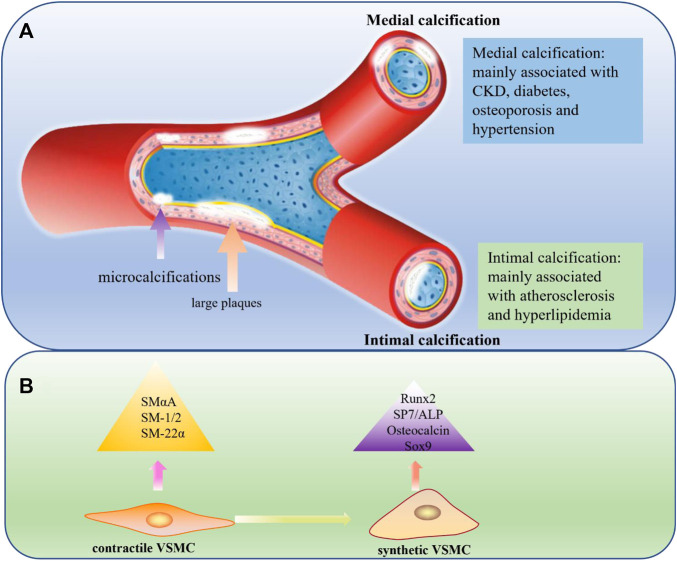
**(A)** Medial calcification and Intimal calcification. In medial calcification, minerals are mainly deposited in the medial layer of blood vessels and usually associated with CKD, diabetes, osteoporosis and hypertension. In intimal calcification, minerals are primarily deposited in the intimal of the blood vessels and develop from microcalcifications to large plaques, usually associated with atherosclerosis and hyperlipidemia. CKD: chronic kidney disease. **(B)** Comparison of contractile VSMC and synthetic VSMC. VSMCs paly an important role in medial calcification and can undergo a phenotypic transition from a physical contractile phenotype to a pathological synthetic phenotype, lossing contractile markers like SMα-A, SMα-22, SM-1 and SM-2 and expressing mineralization-related markers like Runx2, SP7, ALP, osteopontin and Sox9.

In intimal calcification, which is mainly induced by lipid deposition, inflammation, and necrosis, minerals are primarily deposited in the intima of the blood vessels. This often occurs in large arteries, is linked to obstructive arterial disease, and is usually associated with atherosclerosis (Doherty et al., 2003). Intimal calcification occurs over a wide range of patient ages, and with age, the formation of atherosclerotic plaques increases (Abedin et al., 2004). In atherosclerotic lesions, calcification initially appears in the form of microcalcifications (<5 μm) caused by apoptotic or necrotic cells. If this is not controlled, the calcified plaques can gradually progress to large plaques or even rupture, leading to harmful consequences (Roijers et al., 2011). In medial calcification, mineral deposits occur in the medial layer of the vessel wall whose main components are VSMCs and the extracellular matrix full of elastin. Unlike intimal calcification, medial calcification is often associated with CKD, diabetes, hypertension, osteoporosis, and aging (Lanzer et al., 2021). The most important feature of medial calcification is that it is very similar to bone formation processes, because its main cells, VSMCs, lose their original properties and transdifferentiate into osteoblast-like cells through a process regulated by *BMP2*, *MSX2*, *ALP*, and other genes (Iyemere et al., 2006; Shanahan et al., 2011; Leopold, 2015) (Figure 1A).

### Cell Types Involved in Vascular Calcification

As a complex regulated active process, the occurrence and development of VC involves the participation of various cell types, including vascular wall resident cells, stem cells, and progenitor cells from the circulating blood (Cho et al., 2018).

Endothelial cells (ECs) have phenotypic plasticity and can transform into mesenchymal cells through a process that is commonly referred to as the endothelial-mesenchymal transition (EndMT) (DeRuiter et al., 1997; Arciniegas et al., 2007). EndMT enables ECs to differentiate into stem cells that have the potential to form chondrocytes and osteocytes and is closely related to microvascular mineralization (Medici et al., 2010). Further studies have shown that EndMT is the main mechanism involved in the occurrence and development of atherosclerosis and is also a key factor in atherosclerosis, which has an important relationship with mineral deposition and plaque stability (Boström, 2016; Guihard et al., 2016; Wesseling et al., 2018).

VSMCs are mainly located in the middle layer of blood vessels, secrete extracellular matrix, and regulate blood pressure by regularly contracting and relaxing (Dart and Kingwell, 2001). Similar to ECs, VSMCs can also undergo a phenotypic transition, from a physical contractile phenotype to a pathological synthetic phenotype, when stimulated by some factors such as inflammation and oxidative stress (Frismantiene et al., 2018). The most typical characteristic of the osteo/chondrocyte phenotypic switch is the loss of contractile markers like SM α-actin (SMαA), SM-22α, SM myosin heavy chains SM-1 and SM-2 and the upregulation of mineralization-related markers like Runx2, SP7, osteopontin, osteocalcin, and alkaline phosphatase (ALP) and Sox9 (Schurgers et al., 2018). Phenotypic transformation of VSMCs plays a key role in promoting the progression of VC (Durham et al., 2018; Jaminon et al., 2019). Furthermore, prior studies have suggested that abnormal VSMC proliferation and migration plays an important promoting role in atherosclerosis progression (Doran et al., 2008; Wang et al., 2014) (Figure 1B).

Various circulating progenitor cells have been found to be associated with VC, including endothelial progenitor cells (EPCs), mesenchymal stem/progenitor cells, and myeloid cells. In some diseases, such as coronary artery disease and aortic valve stenosis, investigators have found that the proportion of EPCs with an osteogenic phenotype increased (Fadini et al., 2012; Zhang et al., 2015; Yang et al., 2017a; Al-Hijji et al., 2019). Notably, Liao et al. discovered that bone marrow mesenchymal stem cell transplantation after balloon angioplasty in rats with hyperlipidemia resulted in vascular remodeling and calcification (Liao et al., 2012).

### Factors That Cause Vascular Calcification

VC is a highly complex pathological process, and many factors have been found to cause or promote VC. Such discoveries lay the foundation for subsequent drug development. Many factors cause VC, including a variety of cell types, molecules, genes, and environmental factor. For example, extracellular vesicles can be found in calcified aortic media and in atherosclerotic intimal plaques, which suggests a link between the vesicles and VC. Furthermore, calcified extracellular vesicles formed by VSMCs have been found to aggregate with each other to form microcalcification (Hutcheson et al., 2016; Bakhshian Nik et al., 2017). Matrix vesicles belong to extracellular vesicles, and they have been found to play an important role in VC, especially in the early stage (Li et al., 2022a). Duan et al. found that endoplasmic reticulum stress can promote apoptosis, which further accelerated the process of VC and is accompanied by the upregulation of apoptosis markers, such as CHOP and CASP12 (Duan et al., 2009).

Inflammation also plays a role in VC. mRNAs of inflammatory factors, such as Nalp3, ASC, and caspase-1, have been found to be upregulated in calcified VSMCs, while the inhibition of Nalp3 expression by Nalp3KD has been found to block VSMC calcification (Wen et al., 2013). Furthermore, interleukin-6 (IL-6)/soluble interleukin-6 receptor (sIL-6R) complexes, which play a role in the transformation of VSMCs to an osteogenic phenotype, and TNF-α and IL-1β, which participate in the induction of EndMT in human primary aortic ECs, are important inflammatory cytokines that accelerate VC (Kurozumi et al., 2019; Sánchez-Duffhues et al., 2019). In addition, autophagy, apoptosis, osteoporosis, apolipoprotein, and many other factors have been reported to be related to the occurrence and progression of VC (Proudfoot et al., 2000; Sun et al., 2002; Warburton et al., 2007; Dai et al., 2013; Villa-Bellosta and Egido, 2017).

## The Roles of Sirtuins in Vascular Calcification

The regulatory approach of the sirtuin family is a pattern of epigenetic regulation. Epigenetic regulation is an important regulation mode of eukaryotes. It is the structural adjustment at the chromatin level through DNA methylation, histone modification (including methylation, acetylation, phosphorylation, etc.), and small RNA mediation without changing the structure of the DNA sequence, which alters the expression of genes and alters the phenotype of the organism (Goldberg et al., 2007). In the occurrence and development of VC, epigenetic regulation, including histone acetylation, plays an important role (Esteller, 2011). Histone acetylation mainly refers to lysine acetylation catalyzed by lysine acetyltransferase (Weinert et al., 2014). Lysine acetyltransferase includes two types, type A which are located in nucleus and type B which are located in cytoplasm (Li et al., 2020). Lysine acetylation modulates the development of diseases including VC by altering the protein structure or binding affinity with other proteins to alter the function of the corresponding protein (McLendon et al., 2014). For example, the activation of p300, a widely studied lysine acetylase, has been found to upregulate the expression of osteoblast-related genes such as osteocalcin and ALP by increasing the acetylation of histones (H3 and H4) in aortic valvular calcification models (Gu et al., 2019). The opposite process of histone acetylation, histone deacetylation, is catalyzed by lysine deacetylases, which include histone deacetylases (HDACs) and sirtuins (class III HDACs). Although known as histone deacetylases, the sirtuins family can also catalyze non-histone proteins deacetylation, for example, p53 transcription factor, nuclear factor-κB (NFκB), peroxisome proliferator activated receptor (PPAR)and histone acetyltransferase (HAT) p300 (Lu et al., 2020).

**FIGURE 2:**
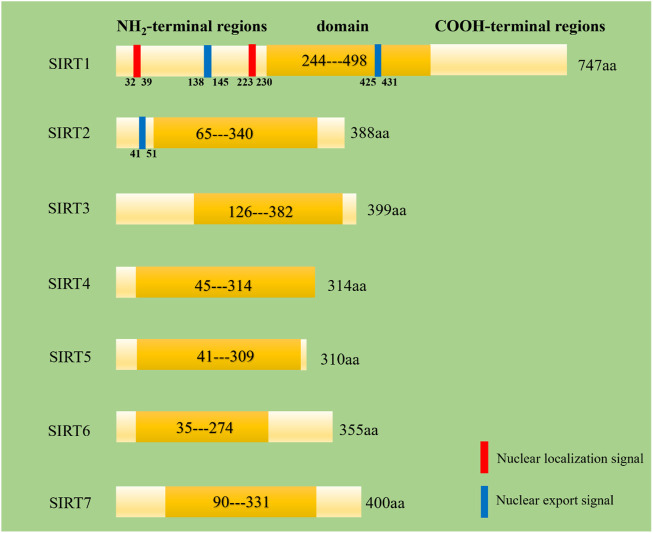
Secondry structures of human SIRT1-7. Domain means a specific combination of secondary structures organized into characteristic three-dimensional structure or fold. These data come from the website http://www.uniprot.org/.

Despite belonging to the same protein family, SIRT1-7 have different amino acid compositions and different structural domains, but all of them have a same highly conserved region (Figure 2) (Jiao and Gong, 2020). What’s more, they have different characteristics in molecular mass, cellular location, enzymatic activity, tissue specificity and biological function (Table 1).

**TABLE 1:** Characteristics of sirtuins including mass, cellular localization, enzymatic activity, tissues and biological functions.

Sirtuins	Mass (Da)^a^	Cellular localization	Enzymatic activity	Tissues^b^	Biological function	References
SIRT1	81,681	Nucleus, cytoplasm	Deacetylase	Low tissue specificity	Regulate oxidative stress and inflammation, aging (life span and health span), calorie restriction/energetics, mitochondrial biogenesis, cellular senescence, endothelial functions, apoptosis/autophagy	(Michishita et al., 2005; Hwang et al., 2013; Singh and Ubaid, 2020; Wang and Chen, 2020)
SIRT2	43,182	Cytoplasm, nucleus	Deacetylase	Enhanced in skeletal muscle, tongue	Neurological function, mitosis regulation, genome integrity, cell differentiation, cell haemostasis, oxidative stress, autophagy	(Michishita et al., 2005; Sayd et al., 2014; Wang et al., 2019b)
SIRT3	43,573	Mitochondria	Deacetylase, Depropionylase	Low tissue specificity	Almost all aspects of mitochondrial metabolism and haemostasis, like urea cycle, TCA cycle, ROS production, apoptosis	(Michishita et al., 2005; Wang et al., 2020; Zhang et al., 2020)
SIRT4	35,188	Mitochondria	Deacetylase, ADP-ribosyltransferase, Lipamidase	Low tissue specificity	Lipid homastasis, Insulin secretion	(Michishita et al., 2005; Haigis et al., 2006; Laurent et al., 2013a; Laurent et al., 2013b; Min et al., 2018)
SIRT5	33,881	Mitochondria	Deacetylase, Desuccinylase, Desmalonylase	Low tissue specificity	Glycolysis, the TCA cycle, fatty acid oxidation, electron transport chain, ketone body formation, nitrogenous waste management	(Michishita et al., 2005; Du et al., 2011; Kumar and Lombard, 2018)
SIRT6	39,119	Nucleus	Deacetylase, Demyristoylase	Low tissue specificity	Heterochromatin stabilization and silencing; stem cell biology; cancer initiation and progression; metabolic homeostasis	(Michishita et al., 2005; Jiang et al., 2013; Tasselli et al., 2017)
SIRT7	44,898	Nucleus	Deacetylase, Desuccinylation	Low tissue specificity	Gene regulation; genome stability; ageing; tumorigenesis	(Michishita et al., 2005; Barber et al., 2012; Li et al., 2016; Blank and Grummt, 2017; Tang et al., 2021)

a These data come from the website https://www.uniprot.org/.

b This information come from the website https://www.uniprot.org/.

### Sirtuin 1

**FIGURE 3:**
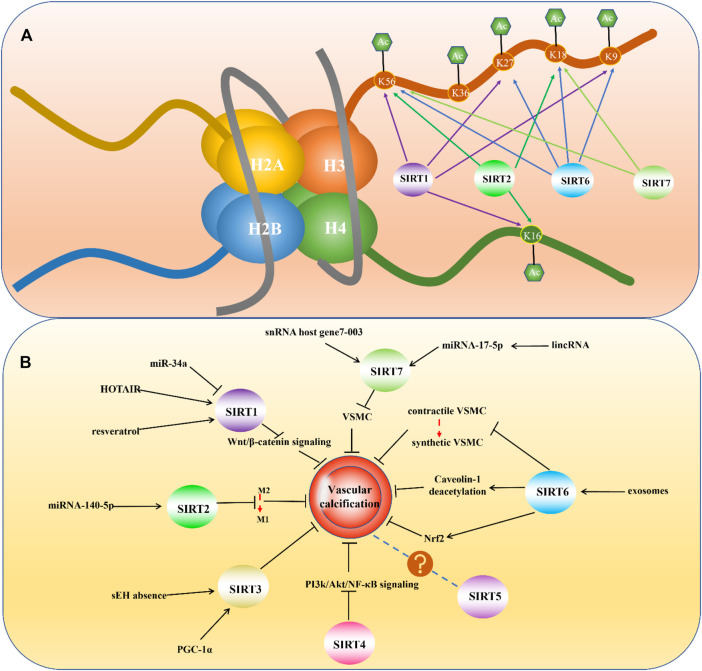
**(A)** Histone deacetylation sites of mammalian Sirtuins. **(B)** Mammalian Sirtuins and their Relevance in Vascular Calcification.

SIRT1 is the first discovered and most well-understood sirtuin. The normal expression of SIRT1 is very important for maintaining physiological function, and many diseases are associated with dysregulated SIRT1 expression, such as cancer, neuroinflammation-related diseases, depression, and cardiovascular diseases (Karbasforooshan and Karimi, 2017; Lu et al., 2018; Alves-Fernandes and Jasiulionis, 2019; Jiao and Gong, 2020). SIRT1 contains 747 residues and consists of four regions: N-terminal domain, allosteric site, C-terminal domain, and catalytic core, which is highly conserved (Huhtiniemi et al., 2006; Autiero et al., 2008). The subcellular localization of SIRT1 can vary in different tissues and species. It is mainly expressed in the nucleus, but also can be found in the cytoplasm (Michishita et al., 2005). As a nuclear sirtuin, multiple studies have demonstrated that SIRT1 can deacetylate histones. Alejandro Vaquero et al. found that in humans, SIRT1 could deacetylate histone H1 at lysine 26 and promote facultative heterochromatin formation. They also proved that SIRT1 could catalyze the deacetylation of H3 at lysine 9 and H4 at lysine 16 (Vaquero et al., 2004). Chandrima Das et al. found that human SIRT1 and SIRT2 could deacetylate histone H3 at lysine 56 (Das et al., 2009) (Figure 3A).

Studies also found important links between SIRT1, histone deacetylation and VC. Francesca et al. conducted a series of experiments to determine whether SIRT1 can protect against DNA damage-induced cell senescence and phenotype transformation of VSMCs in diabetes, they found that loss of SIRT1 in diabetes can accelerate DNA damage, which further exacerbated VC, while SIRT1 activation reduced DNA damage partially by deacetylating around H3K27ac mark within the transcription start site, which further promoted the formation of the active MRN repair complex (MRE11 RAD50, NBS1) (Bartoli-Leonard et al., 2021). A study focusing on the relationship between S-adenosine homocysteine hydrolase (SAAH) and atherosclerotic calcification found that adenosine supplementation activated AMP-activated protein kinase, which further induced SIRT1 expression followed by increase of histone H3 deacetylation and thereby inhibited H19 expression in SAHH-deficient VSMCs, which eventually inhibited osteoblastic differentiation of VSMCs (Dai et al., 2022).

Many other studies also confirmed the protective effects of SIRT1 against VC. Notably, lifelong SIRT1 overexpression in mice has been found to relieve large artery stiffening with advancing age (Machin et al., 2020). Certain molecules can affect VC through SIRT1 signaling. The underlying mechanisms can involve against VC by upregulating SIRT1 expression or accelerate the development of VC by downregulating SIRT1 expression. When investigating whether miR-34a could regulate VC, Ileana et al. reported that miR-34a promoted VSMC calcification by downregulating SIRT1 (Badi et al., 2018). Furthermore, HOTAIR overexpression was found to relieve VC caused by phosphate (Pi) overload by regulating the miR-126/Klotho/SIRT1 signaling pathway. Through this pathway, the increased expression of SIRT1 suppressed Wnt/β-catenin signaling and further inhibited VC (Chen et al., 2021a). And Wnt/β-catenin is well verified to play an important role in osteogenesis calcification and alveolar bone remodeling (Wang et al., 2022). Similarly, SIRT1 signaling has been found to be activate during the resveratrol-induced improvement of aortic calcification in ovariectomized rats (Hammad et al., 2021).

Based on these studies, we believe that SIRT1 is an important cardiovascular protective factor with implications in preventing VC.

### Sirtuin 2

SIRT2 also has important biological functions and has been found to be associated with many diseases, including cancer, neurological disorders, and cardiovascular diseases (Chen et al., 2020a; Chen et al., 2021b; Taneja et al., 2021). SIRT2 is unique because it is the only sirtuin to be mainly distributed in the cytoplasm and can also be found in the nucleus (Vaquero et al., 2006). SIRT2 consists of a 304-amino acid catalytic core and a 19 residue N-terminal helical extension. The core is mainly composed of two domains: the larger one is a variant of the Rossmann fold23, which exists in many different NAD (H)/NADP (H)-binding enzymes, and the smaller one contains a zinc atom (Finnin et al., 2001). Although mainly located in the cytoplasm, under special circumstances SIRT2 can translocate into the nucleus and deacetylase histones. Studies found that during infection, SIRT2 could mediate the deacetylation of histone H3K18 (Eskandarian et al., 2013). During mitosis, SIRT2 can also enter the nucleus and deacetylate H4K16 (Vaquero et al., 2006) (Figure 3A).

Compared with SIRT1, the influence of SIRT2 on VC has not been thoroughly investigated; however, in recent years, several studies on SIRT2 and atherosclerosis have been reported. Liu et al. studied global gene expression changes caused by SIRT2 knockout in primary human umbilical vein ECs (HUVECs) under oxidative stress. The investigators found that SIRT2 knockout altered the expression of 340 genes that participate in many physiological processes and their functions (Liu et al., 2013). Furthermore, Zhang et al. discovered that SIRT2 overexpression prevented high glucose-induced vascular ECs injury by regulating the p53 and NF-κB signaling pathways (Zhang et al., 2018a). These findings indicate that Sirt2 might be associated with atherosclerosis, considering that ECs are closely related to atherosclerosis. Zhang et al. treated female mice whose LDL receptors were knocked out (LDLr−/−) with different conditions and assessed atherosclerotic plaques in the aortic sinus. This investigation revealed that SIRT2 slowed atherosclerotic plaque progression and stabilized the disease condition in LDLr−/− mice. This effect was achieved by inhibiting the differentiation of macrophages into the M1 phenotype (Zhang et al., 2018b). SIRT2 also functions in the signaling pathway through which other molecules affect atherosclerosis. Notably, microRNA-140-5p, an endogenous small non-coding RNAs, was found to relieve hypertension and oxidative stress in atherosclerosis by functioning on SIRT2 and Nrf2 (Liu et al., 2019).

SIRT2 has been consistently shown to protect against VC, especially atherosclerosis. There remains much to learn regarding SIRT2 and its utility as a target for the development of drugs to prevent and treat VC.

### Sirtuin 3

SIRT3 has many important biological functions in endothelial metabolism, angiogenesis, and cardiovascular disease (He et al., 2019). The complete SIRT3 protein is composed of 399 amino acids. There is a mitochondrial targeting sequence composed of 101 amino acids at its N-terminus that is cut off as the protein undergoes activating transformation. SIRT3 contains two domains: the larger one has a Roschmann fold and NAD + binding site, and the smaller one contains a helical complex and a zinc binding group (Jin et al., 2009). Known as mitochondrial sirtuin, human full-length SIRT3 is localized only in the mitochondria, while the short isoform is present in the mitochondria, nucleus, and cytoplasm (Onyango et al., 2002; Scher et al., 2007; Iwahara et al., 2012). Based on existing studies, the effects of SIRT3 on the vasculature are primarily protective (Liu et al., 2021a).

SIRT3 influences both medial calcification, which is closely related to VSMCs, and intimal calcification, which is primarily linked to ECs. Jing et al. used a rat model to explore the correlation between SIRT3 gene expression and EC apoptosis in atherosclerosis. The investigators found that SIRT3 expression was downregulated in the aorta of rats with atherosclerosis and closely related to apoptosis. This finding suggests that SIRT3 has a protective effect on atherosclerosis and may be found to be an important target for preventing atherosclerosis in the future (Jing et al., 2019).

Several recent studies have confirmed the protective role of SIRT3 in VC in patients with CKD. He et al. investigated the role of soluble epoxide hydrolase (sEH) as a potential regulator of VC in CKD. The investigators found that knocking out of sEH slowed the progression of VC linked to CKD through promoting SIRT3 expression (He et al., 2021). Furthermore, Feng et al. discovered the positive function of PGC-1α in CKD-related VC, which involved the restoration of SIRT3 expression and reduction in mitochondrial oxidative stress (Feng et al., 2019).

As SIRT3 research continues, its role in VC will be better understood. Furthermore, SIRT3 may also play an important role in VC prevention and VC treatment.

### Sirtuin 4

SIRT4 is important for the regulation of energy metabolism and mitochondrial function. The abnormal expression of SIRT4 is related to diabetes, liver disease, cancer, heart disease, and many other diseases (Betsinger and Cristea, 2019). Similar to SIRT3, SIRT4 is mainly distributed in mitochondria (Michishita et al., 2005). An increasing number of studies on SIRT4 have been conducted in recent years. However, reports related to VC remain relatively lacking, and the role of SIRT4 in VC remains unclear. It is well known that the most relevant enzymatic activity of sirtuins and VC involves deacetylase activity, however, the deacetylase activity of SIRT4 is weak (Li et al., 2018). This observation does not necessarily imply that SIRT4 has no effect on VC.

Several studies have identified a protective role of SIRT4 in atherosclerosis. For example, Tao et al. explored the function of SIRT4 in atherosclerosis progression. They found that in HUVECs, SIRT4 expression was inhibited by oxLDL treatment, while SIRT4 overexpression enhanced oxLDL-induced HUVEC proliferation induced by oxLDL and curbed cell apoptosis. Further analysis showed that SIRT4 overexpression improved the survival rate of HUVEC cells and reduced the expression of inflammatory factors in HUVEC cells that were induced by oxLDL, by inhibiting PI3K/Akt/NF- κ B signaling (Tao et al., 2019). This study changed our previous view and inspired further investigators to continue to explore SIRT4. It cannot deny SIRT4 as a potential target for developing valuable medicines to prevent or treat atherosclerosis, but the feasibility of SIRT4 as a therapeutic target still needs more research to explore.

### Sirtuin 5

SIRT5 has many significant regulatory roles in normal physiological and pathological processes, particularly in neoplasia (Kumar and Lombard, 2018). The human *SIRT5* gene encodes two major SIRT5 isoforms, SIRT5iso1, which contains 310 amino acids, and SIRT5iso2, which is composed of 299 amino acids. The main difference between them is in their C-termini (Mahlknecht et al., 2006; Matsushita et al., 2011). SIRT5 is mainly localized in the mitochondria (Michishita et al., 2005). Similar to SIRT4, its deacetylation activity is weak even undetectable. However, it has enzymatic activities other than acetylation, such as lysine demalonylation and desuccinylation activities (Du et al., 2011). However, focusing on the studies about SIRT5 and VC, valuable references can’t be found. Even about the connection of SIRT5 to ECs and VSMCs, none related studies have been found. Considering that SIRT5 has almost no deacetylation activity, which is important in vascular biology, it is reasonable to speculate that SIRT5 has little connection with VC. However, with continuous efforts and subsequent in-depth research, it is possible that there will be new discoveries in the future.

### Sirtuin 6

SIRT6 has many functions in regulating lifespan, and abnormal SIRT6 expression has been found to be involved in the pathogenesis of many health-threatening diseases, such as steatohepatitis, diabetes, tumors, neurodegenerative diseases, and cardiovascular diseases (Liu et al., 2021b). Consisting of 355 amino acids, SIRT6 is composed of a putative catalytic sirtuin core and two flanking extensions: a N-terminal extension and a C-terminal extension (Pan et al., 2011). SIRT6 is also situated in the nucleus but is different from SIRT1 in its subnuclear localization (Michishita et al., 2005). It is widely accepted that SIRT6 has deacetylase activity and plays a protective role in VC. Located in nucleus, SIRT6 has been found to deacetylate histone H3 at lysine 9 and regulate telomeric chromatin (Michishita et al., 2008). What’s more, studies also demonstrated SIRT6 could act on lysine 56 on the global core of histone H3 (Michishita et al., 2009). A chemical biology approach also revealed deacetylation of H3K18 and H3K27 by SIRT6 (Wang et al., 2016) (Figure 3A).

Studies have shown that SIRT6 can inhibit VC progression by catalyzing histones. Mandy et al. found that SIRT6 could protect telomeres from damage by deacetylating telomere chromatin at H3K9 and H3K27, thereby preventing VSMCs senescence, which ultimately prevented the occurrence of atherosclerosis (Grootaert et al., 2021).

SIRT6 can affect VC processes, including intimal and medial calcification, by regulating VSMCs, ECs and other cells. In CKD, SIRT6 can inhibit the osteogenic transdifferentiation of VSMCs from a contractile phenotype to a synthetic phenotype, which is the central process in medial calcification, by binding to runt-related transcription factor 2 (Runx2) and causing its deacetylation (Li et al., 2022b). Having known that ECs are important for preventing atherosclerosis, Xu et al. conducted a series of studies that investigated the influence of SIRT6 on ECs, and the results confirmed that SIRT6 played an important role in preventing endothelial dysfunction in mice and the development of atherosclerosis (Xu et al., 2016). A new study showed SIRT6 could act on and deacetylate caveolin-1 in ECs, which activated autophagic degradation of caveolin-1 and inhibited high glucose stimulated LDL transport, which further inhibited the formation of atherosclerosis in diabetes (Zhao et al., 2022). Greiten et al. also confirmed that SIRT6 can prevent oxidative stress, endothelial dysfunction, and vascular dysfunction (Greiten et al., 2021).

The mechanism by which SIRT6 protects endothelial function has also been investigated. Jin et al. studied the role of SIRT6 in minute cholesterol crystals and concluded that SIRT6 could inhibit cholesterol crystal-induced endothelial dysfunction by activating Nrf2 (Jin et al., 2020). EC dysfunction and senescence can be promoted by SIRT6 deficiency, and the mechanism involves the downregulation of forkhead box M1 expression (Lee et al., 2020b). In addition to VSMCs and ECs, the normal expression of SIRT6 in bone marrow-derived cells has functional implications. SIRT6 deficiency in these cells can cause atherosclerosis, in which macrophage scavenger receptor1 plays a central role (Arsiwala et al., 2020). Exosomes are important regulatory structures that have been widely studied in recent years and are involved in the regulation of SIRT6-related signaling pathways. Wang et al. found that exosomes derived from bone marrow mesenchymal stem cells could activate the SIRT6-HMGB1 deacetylation signaling pathway, which restrained high phosphate-induced aortic calcification and protected renal function (Wei et al., 2021).

As an important vascular protective factor, SIRT6 deserves more attention and research, and it is also promising for the development of drugs to prevent or treat VC.

### Sirtuin 7

SIRT7 used to be the least studied sirtuin, but some research breakthroughs have confirmed that SIRT7 has important biological functions and is associated with a variety of diseases, including heart disease, fatty liver, and many types of tumors (Li et al., 2019). SIRT7 is also concentrated in the nucleus, but its subnuclear localization differs from that of SIRT1 and SIRT6 (Michishita et al., 2005). As nuclear sirtuin, study found that SIRT7 were capable of deacetylating histone H3 at lysine 18 and played an important role in maintaining oncogenic transformation of cancer cells (Barber et al., 2012). Besides, H3K36 has been found deacetylating substrates of SIRT7 (Wang et al., 2019a)(Figure 3A). Except for the deacetylation activity, histone desuccinylation activity was also found in SIRT7. More accurately, SIRT7 could desuccinylate histone H3 at lysine 122 and played a role in chromatin compaction and genome stability (Li et al., 2016).

The link between SIRT7, histone deacetylation and VC is not clear, but certain investigations have found that SIRT7 has a protective effect on the progression of VC, especially in atherosclerosis. Zheng et al. explored the function of SIRT7 in regulating the proliferation and migration of VSMCs using an atherosclerosis cell model and concluded that SIRT7 inhibited VSMCs proliferation and migration, which further promoted atherosclerosis by enhancing Wnt/β-catenin activation, and these findings suggest that SIRT7 inhibits the progression of atherosclerosis (Zheng et al., 2018). Certain small molecules have been found to affect atherosclerosis by modulating SIRT7 expression. Notably, Zheng et al. found that the small nucleolar RNA host gene 7-003 can act on the miR-1306-5p/SIRT7 signaling pathway, thereby inhibiting the proliferation, migration, and invasion of VSMCs. This indirectly proves the significant role of SIRT7 in atherosclerosis (Zheng et al., 2021). Furthermore, Wang et al. concluded that p53-dependent lincRNA-p21 could upregulate SIRT7 expression by acting on microRNA-17-5p to prevent VSMCs proliferation and counteract VSMCs apoptosis in atherosclerosis (Wang et al., 2021). In the future, research on SIRT7 needs to be conducted to improve our understanding of the role of SIRT7 in VC development. Whether SIRT7 can be used as a target for the treatment of VC also needs more research to prove.

## Discussion

VC is associated with hypertension, atherosclerosis, and other cardiovascular diseases; however, there are no clinically available measures to treat VC. Therefore, it is necessary to develop drugs that can prevent or reverse the pathological process of VC. Researchers have found that sirtuins play a significant role in maintaining the physiological functions of the vasculature. Sirtuins protect the physiological state of VSMCs and ECs, enabling them to cope with adverse conditions that occur during lipid deposition, oxidative stress, and inflammation, thereby preventing the occurrence of VC. The signaling pathways involved in the molecular link between sirtuins and VC are constantly being elucidated. Based on these findings, some potential drugs have been discovered that modulate the process of VC by changing the expression of sirtuins and regulating related signaling pathways. Liu et al. found that spermidine could inhibit VC in CKD by acting on the SIRT1 signaling pathway, specifically by upregulating the expression of SIRT1 (Liu et al., 2021c). Chen et al. also concluded that Intermedin1-53 could increase the expression of SIRT1 by activating PI3K/Akt, AMPK, and cAMP/PKA signaling, thereby inhibiting the progression of VC related to aging (Chen et al., 2020b). Additionally, Han et al. found that acacetin could resist mitochondrial damage induced by a high-glucose environment, whereas inhibiting the expression of SIRT3 eliminated the protective effect of acacetin. Further analysis showed that acacetin suppressed atherosclerosis aggravated by diabetes may be mediated by activating the Sirt1/Sirt3/AMPK signaling pathway to protect mitochondrial function (Han et al., 2020). Notably, 17b-estradiol was found to inhibit the senescence of ECs and slow down the process of atherosclerosis. However, the underlying mechanism remained unclear until Xiang et al. determined that 17b-estradiol inhibited H_2_O_2_-induced senescence in human umbilical vein ECs by upregulating SIRT3 expression and promoting autophagy, which eventually slowed the progression of atherosclerosis (Xiang et al., 2020).

Although all of SIRTs belong to the sirtuin family, but why the effects of knocking them out are not the same? It is possible that the different consequences are related to their different enzymatic activities. The most well-known enzymatic activity of the sirtuin family is deacetylation, but only SIRT1, SIRT2, SIRT3, and SIRT7 have been identified to have a strong deacetylation activity (Jiao and Gong, 2020). However, SIRT4 was found to have ADP-ribosyltransferase and lipamidase activities (Haigis et al., 2006; Min et al., 2018). SIRT5 was found to possess potent desuccinylation and demalonylation activities (Yang et al., 2017b). The demyristoylating activity of SIRT6 was also found (Jiang et al., 2013). Besides, existing research is mostly based on findings from postnatal studies, but are there some seemingly unimportant sirtuins that actually play an important role during the embryonic period? In other words, are the functions of sirtuins temporally specific? With the continuous advancement of research technology and the continuous exploration of more research, the answer will be clearer.

Although it seems that targeting sirtuins might be effective in VC treatment, whether sirtuin therapeutics can only hold back further progress or even take a turn for the worse calcification remains unclear. In general, the sirtuin family is closely related to VC, and the roles played by different sirtuins are constantly being discovered. Based on the current knowledge, except for SIRT5, other sirtuins play a protective role in VC more or less (Figure 3B). The development of drugs against VC and cardiovascular diseases based on sirtuin is also very promising. In any case, it is undoubtedly important to further understand the influence of sirtuins on VC and the specific mechanisms through which they function. Only after a deeper understanding of the sirtuin family is established will researchers be able to elucidate the best therapeutic targets and develop clinically applicable drugs for the prevention and treatment of VC.
